# Adversity-induced relapse of fear: neural mechanisms and implications for relapse prevention from a study on experimentally induced return-of-fear following fear conditioning and extinction

**DOI:** 10.1038/tp.2016.126

**Published:** 2016-07-19

**Authors:** R Scharfenort, M Menz, T B Lonsdorf

**Affiliations:** 1 Department of Systems Neuroscience, University Medical Center Hamburg-Eppendorf, Hamburg, Germany

## Abstract

The efficacy of current treatments for anxiety disorders is limited by high relapse rates. Relapse of anxiety disorders and addiction can be triggered by exposure to life adversity, but the underlying mechanisms remain unexplored. Seventy-six healthy adults were *a priori* selected for the presence or absence of adverse experiences during childhood (CA) and recent past (RA; that is, past 12 months). Participants underwent fear conditioning (day 1) and fear extinction and experimental return-of-fear (ROF) induction through reinstatement (a model for adversity-induced relapse; day 2). Ratings, autonomic (skin conductance response) and neuronal activation measures (functional magnetic resonance imaging (fMRI)) were acquired. Individuals exposed to RA showed a generalized (that is, not CS− specific) fear recall and ROF, whereas unexposed individuals showed differential (that is, CS+ specific) fear recall and ROF on an autonomic level despite no group differences during fear acquisition and extinction learning. These group differences in ROF were accompanied by corresponding activation differences in brain areas known to be involved in fear processing and differentiability/generalization of ROF (that is, hippocampus). In addition, dimensional measures of RA, CA and lifetime adversity were negatively correlated with differential skin conductance responses (SCRs) during ROF and hippocampal activation. As discriminating signals of danger and safety, as well as a tendency for overgeneralization, are core features in clinically anxious populations, these deficits may specifically contribute to relapse risk following exposure to adversity, in particular to recent adversity. Hence, our results may provide first and novel insights into the possible mechanisms mediating enhanced relapse risk following exposure to (recent) adversity, which may guide the development of effective pre- and intervention programs.

## Introduction

Anxiety- and stress-related disorders are highly prevalent and tend to be persistent.^1^ In particular, high relapse rates represent a major limitation to long-term remission despite effective psychological and pharmacological interventions.^2^ Thus, relapse prevention may represent a promising intervention point for improving long-term therapeutic efficacy.^3, 4, 5, 6^

Relapse risk for trauma-, stressor- and anxiety-related disorders is substantially enhanced by exposure to (life) adversity.^7, 8^ However, the mechanisms contributing to enhanced relapse risk are not yet understood. Relapse can be experimentally modeled in classical conditioning paradigms through the induction of return-of-fear (ROF) following successful extinction training in both animals and humans.^9, 10^ Thereby, during *differential conditioning*, one of two neural cues (the CS+) reliably predicts an aversive event (unconditioned stimulus, US), whereas a second one (CS−) does not. During *extinction*, both the CS+ and the CS− are presented without the US, leading to a waning of the acquired fear response. Importantly, extinction does not erase fear memories, but generates a competing inhibitory memory trace that coexists with the fear memory trace.^11, 12^ Hence, at a later time, insufficient expression of extinction memories upon re-confrontation with the adverse event (that is, in real life: life adversity; in experiments: the reinstatement (RI) US) results in ROF (that is, fear to the conditioned stimuli) in a laboratory model of clinical relapse.^9^ Thereby, RI-induced ROF (for a review in humans^13^ in animals^11^) serves as an experimental model of adversity-induced relapse of fear in which an adverse laboratory event induces ROF.

In healthy humans, differential conditioning protocols yield evidence for RI-induced ROF specifically (or more pronounced) to the CS+ (*differential RI*) in some studies, whereas others demonstrate ROF to both CS+ and CS− to a similar degree (*generalized RI;* for a review Haaker *et al.*^13^). The ability to maintain discrimination under aversive circumstances or in other words whether ROF is specific for the CS+ or generalized to similar CSs (such as the CS−) may be of critical importance, as discrimination during fear acquisition and extinction has been shown to be negatively associated with pathological anxiety^14, 15^ and predictive of resilient responding to stress.^16^ Mechanisms and consequences of individual differences in differentiability of ROF remain, however, largely unexplored to date. Hence, the individual history of life adversity, as established risk factor for the development and relapse of anxiety disorders,^7, 8^ represents a particularly strong candidate. The present study was designed to explore how adversity may become embedded in the brain and ultimately manifest in autonomic (skin conductance), behavioral (subjective ratings) and neural (that is, hippocampal) measures of discriminating safe from danger cues during an experimental model of adversity-induced ROF (that is, RI) in a total of 84 participants pre-selected based on exposure to adversity during childhood (CA) and/or recent past (RA). Exposure to CA and RA was thereby operationalized in both a categorical and a dimensional way.

As deficits in discriminating dangerous from safe stimuli (generalization) is a common hallmark of anxiety disorders,^15^ we expect individuals exposed to life adversity (during childhood or recent past) to display less discriminative (that is, generalized) ROF on an autonomic, subjective level as well as in brain areas of the fear network following adversity-induced ROF in an experimental model of relapse.

Furthermore, the accumulation of environmental adversities ('allostatic load (AL) hypothesis'^17^) has traditionally been considered critical for subsequent development of psychopathologies.^18, 19, 20, 21^ This is commonly operationalized as a sum score of adverse experiences over life irrespective of developmental timing. Recently, however, an alternative concept, the 'mismatch hypothesis',^22, 23^ proposes a mismatch between early (for example, childhood) and later (adult) environment to be pertinent to disease development. Thereby, the environment during early life is thought to prepare the individual for a life within this environment and its challenges ('match'). However, if early and later environment do not match ('mismatch'), acquired strategies may turn out maladaptive. Hence, predictions with respect to risk or resilience differ between both theories particularly with respect to individuals exposed to adversity during both childhood and recent past. Whereas the mismatch approach would predict low risk, the AL would predict highest risk in this group of individuals exposed to adversity during childhood and recent past. Hence, a second exploratory aim of our work was to directly investigate the impact of life adversity and its developmental timing in an experimental model of fear relapse for what we believe is the first time. In addition, we provide the first direct test of the AL and mismatch hypotheses with respect to experimental measures of anxiety in humans.

## Materials and methods

### Participants

Participants were right-handed with normal/corrected-to-normal vision and are free from current or prior psychiatric/neurological disorders (as assessed by the M.I.N.I.^24^). All participants provided written informed consent to the protocol approved by the local ethics committee (Ärztekammer Hamburg (General Medical Council Hamburg)) and the study was conducted in accordance with the Declaration of Helsinki. In total, 84 participants were recruited from a large pool of 392 participants. This pool of participants served as a 'screening sample' in order to provide well-phenotyped participants (for example, with respect to life adversity and other traits assessed by questionnaires) for studies within the framework of the Collaborative Research Center SFB TRR 58 (refs 25, 26)), which the current study is part of. Three participants had to be excluded from the study (technical issues_day1_ (*N*=1) on day 1; pathological anatomy (*N*=1). An additional five participants had to be excluded from day 2 (drop-out (*N*=1), technical issues (*N*=4)), leaving *N*=76 for analyses.

### Quantification of life adversity

For the present study, participants were *a priori* selected from the screening sample based on the presence or absence of adversity during childhood (CA+/CA−) or recent past (RA+/RA− that is, past 3 years, see Table 1 for sample descriptives and details). Adversity during both time periods was assessed by a modified version of the life events' checklist^27, 28^ that also recorded age of occurrence as well as valence (positive, negative and indifferent) for each of the 27 items (multiple occurrences allowed). For the purpose of this study, only events that were subjectively evaluated as negative were considered (Supplementary Table 1 provides a complete list of reported events) and the experimenter was blinded with respect to this. Methods, materials and procedures are briefly summarized in Table 2 and are described in our previous publication,^29^ which for the first time establishes the neural correlates of RI-induced ROF, which is critical for the interpretation of the present data but could not be included in the present manuscript because of space constraints.

For additional analyses targeting the effect of AL, participants were grouped according to the cumulative number of reported life events throughout lifetime (AL). Exploratory analyses with respect to the mismatch hypothesis are presented in the Supplementary Materials as analyses considering four different (mis-)match groups CA−/RA− (*N*=21, 12 females), CA+/RA+ (*N*=22, 11 females), CA−/RA+ (*N*=20, 11 females) and CA+/RA- (*N*=13, 7 females) in the study sample. For dimensional measures of adversity, the number of individual events during childhood and recent past were considered.

### Procedure

The procedure included intensity calibration of the electrotactile US (mean intensity(s.d.): 6.9(4.9) mA, see Table 1 for group-specific values), habituation and uninstructed acquisition on day 1. Two grey (RGB (230,230,230) snow fractals on a grey background (RGB (100,100,100)) served as the CSs. About 24 h after conditioning (day 2), participants returned to the laboratory for the uninstructed delayed extinction^32, 33^ and subsequent RI and RI-test session. Table 2 provides specific experimental details on all experimental phases (see also Scharfenort and Lonsdorf^29^). All phases were performed within the MR Scanner.

### fMRI data acquisition and analysis

fMRI data were acquired on a 3-Tesla MR-scanner (MAGNETOM trio, Siemens, Erlangen, Germany) using a 32-channel head coil using an echo planar image sequence (repetition time (TR)=2460 ms, echo time (TE)= 26 ms). For each volume, 40 slices with a voxel size of 2 × 2 × 2 mm (1 mm gap) were acquired sequentially. Structural images were obtained by using a T1 MPRAGE sequence. Functional magnetic resonance imaging (fMRI) data were analysed using SPM8 (Welcome Trust Centre for Neuroimaging, University College London, London, UK). Preprocessing included co-registration to the individual structural image, re-alignment, normalization to group-specific templates created via the DARTEL algorithm^34^ as well as smoothing (6-mm full width at half maximum).

As the RI-test phase was of primary interest, we refer to the Supplementary Methods and our previous publication^29^ for a detailed description of the first levels for acquisition and extinction (early/late). For the RI-test, four effect-of-interest regressors were built at the first level (that is, last three extinction and first three RI-test trials for CS+ and CS−) as well as eight nuisance regressors (RI USs; ratings; six movement parameters derived from re-alignment). All regressors of interest were modeled as stick function and time-locked to stimulus (CS/US/rating) onset for volumes of interest (onset of the first regressor of interest-1 × TR until the onset of the last regressor of interest+3 × TR). Regression coefficients (beta values) for the regressors in each voxel were computed via the general linear model. Contrasts of interest (CS+>CS− CS+<CS−) were estimated on the first level and taken to the second-level analyses employing two-sample *t*-tests or a full-factorial model. Significant group differences in CS discrimination (CS+>CS−) at a neural level were further tested by separate analyses for the CS+ and CS−.

To investigate dimensional effects of life adversity, regression analyses were performed in SPM between reported adversity (CA, RA and AL (both CA and RA)) and CS− discrimination (CS+>CS−) for acquisition, early/late extinction and RI separately.

Region of interest (ROI) analyses were based on key areas implicated in RI (amygdala, ventromedial prefrontal cortex (vmPFC), hippocampus, anterior insula cortex (AI), anterior cingulate cortex (ACC) and thalamus^29, 35^). Amygdala, ACC, thalamus and hippocampus masks were available from the Harvard–Oxford cortical and subcortical structural atlases^36^ (threshold: 0.7). The anterior AI mask and a vmPFC mask are not available from this atlas and were thus created by merging the dorsal and ventral AI masks from Deen *et al.*^37^ for each hemisphere separately and by employing a 10-mm sphere centered on vmPFC coordinates derived from an independent study on RI (*x*,*y*,*z*: 0, 40, −12 (ref. 35)).

Familywise error correction was applied to correct for multiple comparisons, and additional whole-brain analyses (*P*<0.00 11µc) are included for completeness and future hypothesis generation. This is provided in the Supplementary Material but not discussed, as is commonly done in fMRI research reports.

### Skin conductance responses

Skin conductance responses (SCRs) were measured using two disposable Ag/AgCl electrodes (2 cm diameter) attached to the distal and proximal hypothenar of the left hand. The signal was recorded using a BIOPAC MP-150 amplifier and Acqknowledge 3.9 software (BIOPAC Systems, Goleta, CA, USA). MR-compatible equipment was used. Data were downsampled to 10 Hz and semimanually scored offline using a custom-made program according to published recommendations,^38^ that is, the first response initiating within 0.9–4.0 s post stimulus (US/CS) onset with a minimum amplitude >0.02 μs. Reactions showing recording artifacts were treated as missing data points and those with an amplitude <0.02 μs were scored as zero responses. Data were log-transformed^39^ and range-corrected to account for inter-individual variability.^40^ SCR data from some participants had insufficient data quality and were therefore excluded from the analysis (*N*_Day1_=1; *N*_Day2_=5).

### Statistical analyses

Data were analyzed using SPSS 22 for Windows (IBM, Armonk, NY, USA), a level of *P*<0.05 (two-sided) was considered as significant and Greenhouse–Geisser-corrected degrees of freedom were used when appropriate.

Statistical analyses for SCRs and subjective ratings were performed for the acquisition, for the first half (that is, fear recall), and second half of extinction separately using repeated-measure analyses of variance with the within-subject factor CS type (mean of the CS+/CS−). RI-test effects^13, 29^ for SCR and subjective ratings were analysed with a repeated-measure analysis of variance with the within-subject factor CS type (CS+/CS−) and time (late extinction and early RI-test). As commonly done for ROF studies, a mean of three single trials per CS type was used for both late extinction and early RI-test in SCRs,^13, 29^ whereas single ratings were used for rating analyses as they were only provided intermittently.^13^ Adversity group (either CA (+/−) or RA (+/−)) served as between-subject variable. Significant main effects of or interactions with group were further investigated with appropriate *post hoc* tests.

Furthermore, to explore dimensional effects of life adversity (CA, RA and AL), regression analyses were performed between reported adversity and CS− discrimination in SCRs and ratings for all experimental phases separately. Data were also explored with respect to a possible mismatch approach (four mismatch groups).

Generally, in the text, we restrict ourselves to reports of fMRI results that have a correspondence in autonomic or subjective measures.

## Results

### Main effect of task

We refer to our previous work for a detailed description of the main effects of task (for example, successful fear acquisition, extinction and ROF) in this sample irrespective of exposure to adversity.^29^ On the basis of our main hypotheses, we focus on the impact of adversity on ROF in this publication.

### Association of recent and childhood adversities with fear acquisition, extinction and fear recall

**Figure 1:**
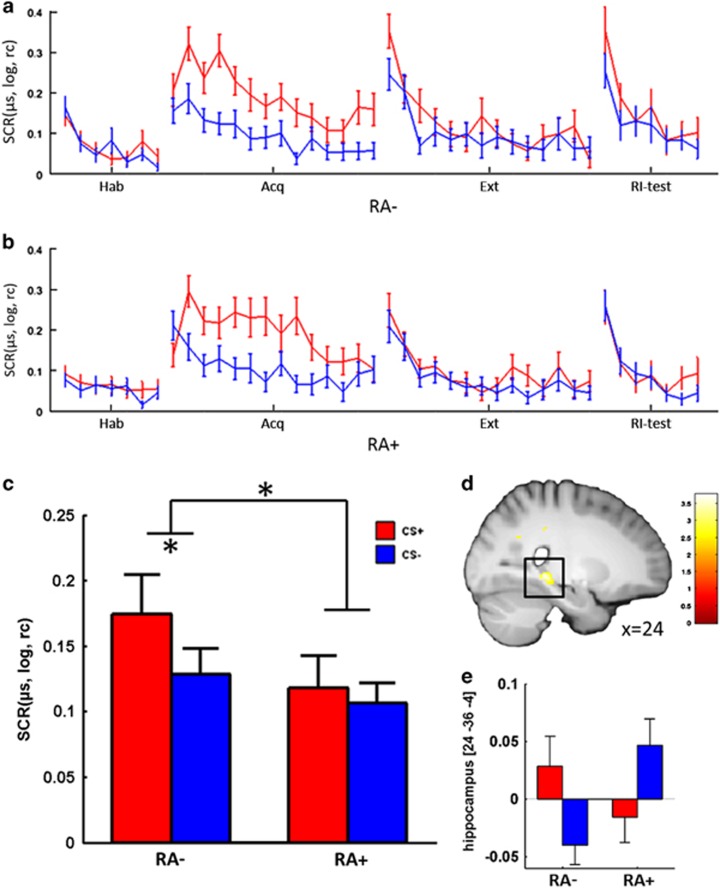
(**a–e**) Skin conductance response (SCR) and neural activation reflecting group differences in CS− discrimination during fear recall. (**a**) Log-transformed (log) and range-corrected (rc) SCR (in μs) during all experimental phases for individuals not exposed to (**a**) and exposed to (**b**) recent adversity. SCRs during fear recall (that is, early extinction) for groups without and with exposure to recent adversity (RA− and RA+, respectively). (**c**) Neural activation reflecting group differences in CS− discrimination during fear recall (RA−_Ext1(CS+>CS−)_>RA+_Ext1(CS+>CS−)_) on a visualization threshold of *p*_uc_<0.01 and (**d**) corresponding beta values (for illustrative purposes; **e**). Error bars represent the s.e.m. *P<0.05. CS, conditioned stimulus.

The impact of adversity on preceding experimental phases (fear acquisition, fear recall and extinction) is presented for completeness in the Supplementary Material. Briefly, exposure to RA was linked to differences in CS− discrimination in both autonomic and neural measurements only during fear recall, but not in any other experimental phase. More precisely, individuals exposed to RA did not show any CS+/CS− discrimination, as indexed by SCR (Figures 1a–c), during fear recall, whereas unexposed individuals showed a differential fear recall. These group differences were mirrored in CS− discrimination differences in the posterior hippocampus (Figures 1d and e). Significant correlations with CA were not observed in any experimental phase or dependent measure (Supplementary Tables 2 and 6 for RA and CA).

### Association of recent adversity with RI-induced ROF

**Figure 2:**
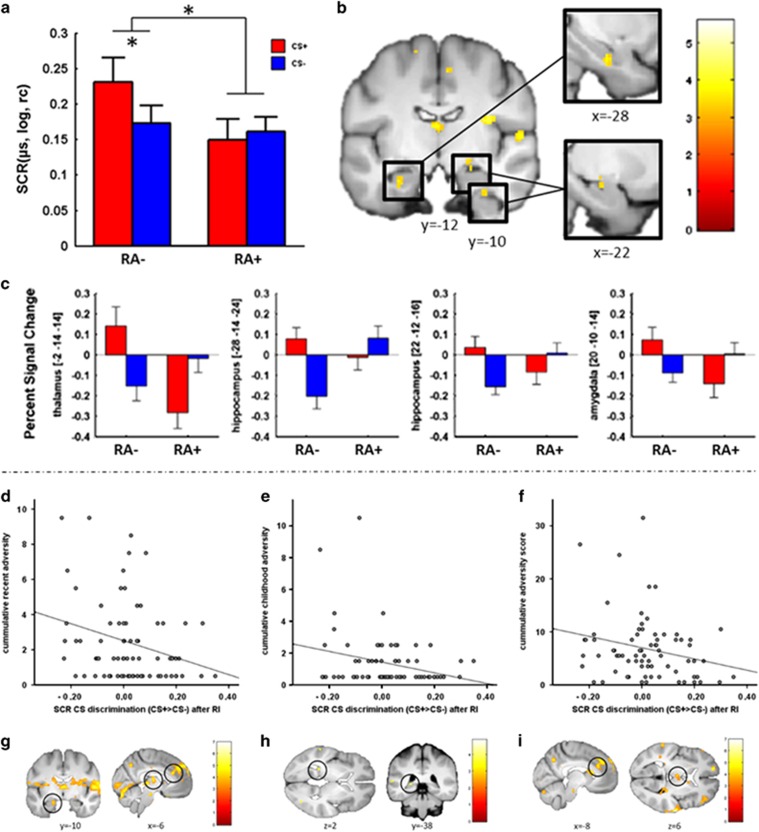
(**a**–**c**) Skin conductance responses (SCRs) and neural activation reflecting group differences in CS− discrimination during reinstatement (RI) test. (**a**–**f**) Cumulative adversity and CS− discrimination in SCR and neural activation during RI-test for dimensional reported recent adversity (RA), childhood adversity (CA) and lifetime adversity (AL). (**a**) The logarithm of the data was taken (log) and range-corrected (rc) mean SCR responses (in μs) for the first three RI trials for groups with and without exposure to recent adversity (RA+ and RA−, respectively). (**b**) Neural activation reflecting differences between groups without and with exposure to recent adversity (RA− and RA+, respectively) in CS− discrimination during RI-test (RA−_RI(CS+>CS−)_>RA+_RI(CS+>CS−)_) on a visualization threshold of *p*_uc_<0.001 and (**c**) extracted beta values (for illustrative purposes) for each of the regions of interest (ROI) with significant group differences. Scatterplots of CS− discrimination (CS+>CS−) in SCR and neural activation for the first three trials after RI for cumulative (**d,** **g**) recent adversity, (**e**, **h**) childhood adversity and (**i**, **h**) adversity throughout life. The visualization threshold is *p*_uc_<0.001. Error bars represent the s.e.m. *P<0.05. CS, conditioned stimulus.

SCRs showed a general increase after RI, as indicated by a significant main effect of time (F(1,69)=45.03, *P*<0.001, *η*^2^=0.39; generalized RI), in absence of a significant time × RA group interaction (F<1.1). In addition, a significant time × stimulus × RA group interaction (F(1,69)=4.0; *P*=0.049; *η*^2^=0.055) indicative of group differences in differentiability of the RI effect was revealed. Those differences were driven by a significant CS type × RA group interaction after (F(1,70)=5.0, *P*=0.027, *η*^2^=0.07, Figure 2a; mainly driven by differences in CS+ responsivity), but not before RI (F(1,70)<1) in absence of a main effect of group (F<1.5). Importantly, whereas significant CS+/CS− discrimination after RI was observed in the non-exposed individuals (RA−: F(1,33)=6.2, *P*=0.018, *η*^2^=0.16), no discrimination was observed in individuals exposed to recent adversity (RA+: F<1). This significantly stronger autonomic CS− discrimination (CS+>CS−) in the RA− as compared with the RA+ group was accompanied by significant ROI-based differences in areas related to the (return of) fear network such as the right amygdala, the left hippocampus and the left thalamus (Table 2 and Figures 2b and c). Importantly, the hippocampus, amygdala and thalamus have shown to be activated during RI-induced ROF previously in this^29^ and other samples.^35^ At an exploratory threshold of *P*<0.001, additional activation differences were observed in frontal, temporal and cerebellar regions (Supplementary Table 5). In line with non-differential SCR responses during ROF, individuals exposed to recent adversity (RA+) did not display stronger differential activation (CS+>CS−) than the RA− group in any ROI.

In contrast to SCRs, no significant interaction with the RA group was observed for subjective fear ratings during RI. Individuals exposed to RA (RA+), however, showed trendwise higher fear ratings than non-exposed (RA− F(1,64)=3.21;*P*=0.078, *η*^2^=0.05 (Supplementary Table 2)).

### Association of childhood adversity with RI-induced ROF

In contrast to recent adversity, no main effect of or interaction with the CA group were observed on SCRs, subjective ratings or in any of the ROIs during ROF (Supplementary Tables 6 and 8).

### Dimensional analyses of childhood and recent adversity as well as AL

Explorative analyses considering dimensional measures of CA and RA were significantly negatively correlated with SCR CS− discrimination during the RI-test, whereas no significant correlation was observed for subjective ratings (Figures 2d and e; Supplementary Tables 3 and 7). Matching autonomic data, a significant negative correlation was observed between dimensional measures of RA and CS− discrimination in the left hippocampus as well as a trend in the right hippocampus (Figure 2g and Table 3). In addition, dimensional measures of RA were negatively correlated with CS− discrimination in the bilateral thalamus and bilateral ACC (albeit only trendwise in the right ACC; Figure 2g and Table 3). Furthermore, a positive correlation between dimensional CA measures and CS− discrimination in the right hippocampus was observed (Figure 2h, Table 3).

Explorative analyses considering a dimensional measure of adversity throughout life (AL) revealed a negative correlation between AL and CS− discrimination in SCRs during the RI-test, although no significant correlation was observed for subjective ratings or in any other experimental phase (Figure 2f and Supplementary Table 9). Convergently, fMRI analyses also reveal a significant negative correlation between AL and CS− discrimination in the bilateral ACC and bilateral thalamus as well as a trend in the right hippocampus during the RI-test (Figure 2i and Table 3), largely mirroring results for recent but not early adversity.

## Discussion

The present work provides converging multimodal evidence from autonomic and neural measures for the impact of categorical as well as dimensional measures of life adversity on the differentiability of fear recall and ROF. Thereby, in particular, recently experienced adversity was associated with RI-induced ROF in an experimental model of adversity-triggered relapse.

More precisely, exposure to recent adversity but not childhood adversity specifically attenuated autonomic CS+/CS− discrimination during unprobed 24-h-delayed fear recall and experimentally induced ROF in absence of discrimination differences during fear acquisition and extinction. These physiological differences in CS discrimination during fear recall and ROF, based on exposure to recent adversity, were nicely mirrored in diminished CS discrimination in brain areas previously linked to fear learning, fear expression and ROF (that is, amygdala, hippocampus and thalamus). Our data thus support that recent exposure to adversity, which is an established risk factor for the development and relapse of affective pathology,^41^ may promote the generalization of fear response during 24-h-delayed fear recall as well as during RI-induced ROF possibly through facilitation of fear memory consolidation.

The ability to maintain discrimination between safety and danger cues under aversive circumstances is of critical importance and has been found to be negatively associated with pathological anxiety,^14^ and predictive of resilient responding to life stress.^16^ Our data thus lend support for the detrimental consequences of exposure to (recent) adversity on affective processing and for the first time provide evidence for individual difference factors contributing to the quality of fear recall, experimentally induced ROF, as well as the underlying neural and mechanistic (that is, fear memory consolidation) underpinnings.

Critically, while patients suffering from anxiety disorders have been shown to display deficient CS− discrimination already during fear acquisition and extinction,^14, 15^ the impact of recently experienced adversity in our study was specific to fear recall and ROF. As ROF following RI has, however, not yet been investigated in clinical populations and such studies are eagerly awaited to show weather experimentally induced ROF might be generally more pronounced or more or less differential in patients than in controls. In light of differences in CS− discrimination being primarily driven by differences in CS+ responsivity in the present study, an alternative explanation, despite deficient discrimination/overgeneralization might be blunted responses to challenges, such as the CS+, as shown for cortisol reactivity in individuals exposed to life adversity.^42^ Even though exposure to adversity increases the risk of clinical relapse, more differential (CS+-related) responding in unexposed individuals in our experiment may also signal higher relapse risk in this group. Even though our previous work suggests that generalized RI (as in the RA+ group) may at least partly be driven by genuine association-based processes,^29^ future studies in patients are needed to extend these first exciting findings. Of note, maltreated children have recently been shown to show blunted CS+ responding in SCRs during fear acquisition as compared with not maltreated children,^43^ which supports the interpretation of our data even though this study did not include a ROF manipulation.

Whereas our results from experimental fear conditioning, extinction and ROF as laboratory models for the acquisition, treatment and relapse of fear identify *recently* experienced life events as a potential major risk factor for the maintenance and the relapse of trauma-, stressor- and anxiety-related disorders, it is a recent debate how different timings of exposure to adversities interact.^25^ Traditionally, the 'AL' hypothesis assumes that an accumulation of adversity over the lifetime increases the vulnerability for affective disorders. More recently, however, this view has been challenged by the assumption that the impact of adversity on brain and behavior may depend on age at exposure.^44^ As such, the '(stress-coping) mismatch hypothesis'^22, 23^ assumes that vulnerability derives from a mismatch between (coping) abilities acquired during early life and the challenges exposed to in later life ('mismatch'). In line with our work on the impact of adversity on anxious temperament and brain morphology,^25^ the present study highlights the specific role of the proximity in timing of negative events and AL rather than supporting a mismatch approach for fear recall and ROF—at least in the present operationalization. Although we do not observe an association between childhood adversity on fear conditioning, extinction and ROF, others have recently reported amygdala-related differences during fear conditioning depending on childhood adversity using Granger causality methods.^45^ Additional work with a more fine-grained operationalization of adversity is, however, warranted as very recent rodent work suggests a possible role of the controllability of the stressor.^46^

Our work represents the first investigation on the role of life adversity and its timing on fear conditioning, extinction and ROF in healthy adults. Importantly, despite of the impact of recent adversity, our data seem to primarily support the AL hypothesis. However, the impact of cumulative adversity throughout life seems to be largely driven by exposure to recent adversity. Our data thus support that recent adversity becomes biologically embedded through changes in neural functioning of limbic areas, which are both highly sensitive to the physiological effects of stress^44^ and implicated in affective psychopathology^47^ as well as the acquisition, recall and ROF.^48^

Although we present intriguing and converging evidence for individual differences contributing to the maintenance of discriminating safe from dangerous stimuli in face of adversity, some limitations of our work should be acknowledged. First, the correlative nature of the analyses of exposure to adversity on experimental read-outs does not allow any causal inferences. Second, because the associations with adversity may, in fact, depend on age of exposure,^44^ it is possible that our results in young adults cannot be generalized to older cohorts. Third, data on exposure to adversity were acquired retrospectively. Even though inevitably in most cross-sectional studies, retrospective reports may inherently suffer from memory and reporting biases.^49^ Fourth, the questionnaire employed in this study is not specifically validated for capturing childhood, and other measures of childhood trauma (such as the Childhood Trauma Questionnaire^50, 51^) might be more sensitive to the impact of early adversity/trauma. However, considering additional information provided by the childhood trauma questionnaire,^50, 51^ which was also available in our sample, no significant associations of CA on RI-induced ROF were observed (data not shown). Fifth, as participants were pre-selected with respect to life adversity (RA/CA) and matched with respect to age/sex across groups, the group of individuals included in here may not be representative of the general populations.

Taken together, our data not only suggest that adversity is associated with the quality (that is, CS− discrimination) of fear recall and RI-induced ROF but also provides novel insights into the possible neural and psychological mechanisms mediating this association. As such, our results have strong clinical implications and may aid the development of novel intervention and prevention programs. Relapse frequency might in fact be reduced through intervention programs specifically targeting discriminating threat from safety as well as promoting de-generalization in particular during the aftermath of traumata or other adverse experiences in remitted patients.

## Figures and Tables

**Table 1:** Characteristics of participants selectively invited based on presence (+) and absence (−) of CA (until the age of 11 years) and RA (past 3 years)^a^

	*RA+*^b^	*RA−*b	*Statistics*	*CA+*^c^	*CA−*c	*Statistics*
*N*	42	34	*Χ*^2^, *P*=0.96	35	41	*Χ*^2^, *P*=0.51
Age (s.d.)	24.2 (3.3)	25.7 (3.6)	F(1,73)=2.94, *P*=0.09	24.6 (3.6)	25.2 (3.4)	F(1,73)<1, *P*=0.38
Female	22	19	*Χ*^2^, *P*=0.96	17	23	*Χ*^2^, *P*=0.51
STAI-S (s.d.) day 1	32.74 (5.3)	35.90 (7.5)	F(1,72)=3.10, *P*=0.08	34.83 (7.0)	34.09 (6.5)	F(1,72)<1, *P*=0.56
STAI-S (s.d.) day 2	31.59 (5.1)	34.98 (6.1)	F(1,72)=5.39, *P*=0.02	33.41 (5.5)	33.51 (6.3)	F(1,72)<1, *P*=0.99
STAI-T	37.75 (10.5)	32.12 (8.2)	F(1,69)=5.37, *P*=0.02	34.53 (9.1)	36.03 (10.9)	F(1,69)<1, *P*=0.50
NEO-FFI neuroticism	29.78 (7.9)	25.35 (6.8)	F(1,71)=5.797, *P*=0.01	27.68 (8.0)	27.88 (7.5)	F(1,71)<1, *P*=0.91
US intensity	6.27 (5.3)	7.51 (4.4)	F(1,72)<1, *P*=0.44	6.64 (3.6)	7.03 (6.1)	F(1,72)<1, *P*=0.68

Abbreviations: CA, childhood adversity; NEO-FFI, NEO Five-Factor Inventory; RA, recent past adversity; STAI-S,

State-Trait Anxiety Inventory - state; STAI-T, State-Trait Anxiety Inventory - trait; US, unconditioned stimulus.

a Presence of adversity was defined as the reported experience of one or more adverse event(s); for more details regarding the reported events see Supplementary Table 1.

b Irrespective of CA.

c Irrespective of RA.

**Table 2:** Summary of methodological details and materials

	Study characteristics
*N* (whereof female)	76 (41)
Mean age (s.d.)	25 (3.5)
Reimbursement	50€
Day 1 (trials per CS type)	Habituation (7), acquisition (14)
Day 2 (trials per CS type)	Extinction (14), RI-test (7)
Visual material CS	2 Gray fractals (340 × 320 pixel) × presented for 6–8 s (mean: 7 s)
Visual material ITI	White fixation cross-presented for 10–16 s (mean: 13 s)
US type	Digitimer DS7A constant current stimulator (Digitimer, Elwyn Garden City, UK) to the back of the right hand with a 1-cm diameter surface electrode with a platinum pin (Specialty Developments, Bexley, UK). It consisted of three 10-ms rectangular pulses with an interpulse interval of 50 ms
Reinforcement ratio	100%
RI background	Cue background (grey screen without fixation cross)
*N* RI USs	3
Time gap last CS_ext_−first US_ri_	30 s
Time gap between USs	5 s
Time gap last US_ri_−first CS_ri-test_	13 s
Time gap RI context onset and RI	5 s
Dependent measures	SCR, BOLD response (fMRI), ratings
Subjective ratings	Fear/stress/tension ratings (most recent encounter^a^) on a 25 stepped VAS (anchored at 0 and 100), retrospectively after each experimental phase
Questionnaires	STAI-S, STAI-T^30^ and NEO-FFI^31^

Abbreviations: BOLD, Blood-oxygen-level dependent; CS, conditioned stimulus; fMRI, functional magnetic resonance imaging; NEO-FFI, NEO Five-Factor Inventory; RI, reinstatement; SCR, skin conductance response; STAI-S, State-Trait Anxiety Inventory - state; STAI-T, State-Trait Anxiety Inventory - trait; US, unconditioned stimulus; VAS, visual analogue scale.

a Except for after RI-test where also the first presentation after RI was retrospectively rated.

**Table 3:** Neural activation reflecting group differences in CS− discrimination (CS+>CS−) during RI-test separately for categorical and dimensional for (the number of) reported RA, CA and lifetime adversity

*Contrast RI*_*CS+>CS−*_	*Brain area*	x	y	z	k	T	p *(µc)*	p*(svc*_*FWE*_)
*Categorical*								
RA−>RA+^a^	Thalamus (L)	−2	−14	14	12	4.30	<0.001	0.010
	Hippocampus (L)	−28	−14	−24	16	3.76	<0.001	0.026
	Hippocampus (R)	22	−12	−16	1	3.34	<0.001	0.080
	Amygdala (R)	20	−10	−14	4	3.39	<0.001	0.038
RA−<RA+^a^	None							
CA−>CA+	None							
CA−<CA+	None							
								
*Dimensional*								
Neg. ass. with AL	Thalamus (L)	−4	−10	6	31	4.38	<0.001	0.008
	Thalamus (R)	6	−18	14	29	3.76	<0.001	0.048
	ACC (L)	−8	30	22	7	4.29	<0.001	0.006
	ACC (R)	6	36	22	3	3.80	<0.001	0.034
	Hippocampus (R)	22	−38	4	1	3.35	0.001	0.080
Pos. ass. with AL	None							
Neg. ass. with RA	Thalamus (L)	−2	−12	6	201	5.14	<0.001	0.001
		−6	−28	2	71	5.04	<0.001	0.001
	Thalamus (R)	2	−12	8	229	4.76	<0.001	0.002
	ACC (L)	−6	28	24	18	4.61	<0.001	0.002
	ACC (R)	6	36	22	2	3.57	<0.001	0.064
	Hippocampus (L)	−18	−10	−20	18	4.43	<0.001	0.004
	Hippocampus (R)	24	−14	−24	4	3.36	0.001	0.080
	Amygdala (R)	22	−12	−12	2	3.33	0.001	0.046
Pos. ass. with RA	None							
Neg. ass. with CA	Hippocampus (L)	−22	−38	2	1	3.55	<0.001	0.047
Pos. ass. with CA	Hippocampus (R)	32	−12	−18	2	3.76	<0.001	0.028

Abbreviations: ACC, anterior cingulate cortex; AL, allostatic load; CA, childhood adversity; CS, conditioned stimulus; neg. ass., negative association; none, no suprathreshold clusters; pos. ass., positive association; RA, recent past adversity; RI, reinstatement.

a For an exploratory whole-brain analysis see Supplementary Table 5.
