# Supplementary material for: Adversity-induced relapse of fear: neural mechanisms and implications for relapse prevention from a study on experimentally induced return-of-fear following fear conditioning and extinction
Source: Transl Psychiatry. 2016 Jul 19;6(7):e858–. doi: 10.1038/tp.2016.126 (PMC5545712; doi:10.1038/tp.2016.126)
Supplement: Supplementary Information [file tp2016126x1.docx]

Supplementary Material for

**Adversity-induced relapse of fear – Mechanisms and implications for relapse prevention from a study on experimentally induced return-of-fear following fear conditioning and extinction**

Robert Scharfenort, Mareike Menz & Tina B. Lonsdorf

**1. Supplementary method**

**1.1. fMRI processing for acquisition and extinction**

First levels were created for acquisition and extinction seperatly. For acquisition two effects-of-interest regressors were built (i.e. CS+ and CS-) as well as eight nuisance regressors (RI-USs; ratings; six movement parameters derived from realignment). For extinction four effects-of-interest regressors were built (i.e. first half or extinction and second half of extinction for CS+ and CS-) as well as seven nuisance regressors (ratings; six movement parameters derived from realignment). All regressors of interest were modeled as stick function and time locked to stimulus (CS/US/rating) onset. Regression coefficients (beta values) for the regressors in each voxel were computed via the general linear model. Contrasts of interest (CS+>CS- and for completeness the inverse CS+<CS-) were estimated on the first level and taken into the second level analyses employing two-sample t-tests. Significant group differences in CS discrinination at a neural level were further tested for the CS specifity of the effect by performing analyses for CS+ and CS- seperatly.

**2. Supplementary Results**

| **2.1. Quantification of life adversity**  **Supplementary Table 1.** Number of reported negative events for each questionnaire item (1-27) of a modified version of the life events checklist^(1)^ separately for recent and childhood adversity. Dashes indicate that the item was not considered. | | | | | | | | | | | | | | | | | | | | | | | | | | | |
| --- | --- | --- | --- | --- | --- | --- | --- | --- | --- | --- | --- | --- | --- | --- | --- | --- | --- | --- | --- | --- | --- | --- | --- | --- | --- | --- | --- |
|  | 1 | 2 | 3 | 4 | 5 | 6 | 7 | 8 | 9 | 10 | 11 | 12 | 13 | 14 | 15 | 16 | 17 | 18 | 19 | 20 | 21 | 22 | 23 | 24 | 25 | 26 | 27 |
| RA+ | 6 | 1 | 0 | 6 | 1 | 1 | 2 | 18 | 29 | 5 | 23 | 2 | 12 | 3 | 6 | 28 | 28 | 20 | 0 | 5 | 1 | 2 | 8 | 0 | 4 | 0 | 20 |
| CA+ | - | - | - | 7 | 0 | 8 | 3 | 1 | 12 | 0 | 14 | 0 | 10 | 8 | 30 | - | - | 2 | - | 0 | - | 0 | 2 | - | 0 | 1 | 2 |
| 1 job loss 15 ongoing disputes between family members  2 traumatic events related to the working place 16 break up with a partner or a friend  3 wedding plans 17 ongoing disputes with the partner  4 house purchase, -selling or move 18 serious problems in a friendship  5 extended renovation 19 enrolment of a child  6 commit a theft or vandalism 20 intensive nursing of and old or ill person  7 infringement 21 abortion for yourself or your partner  8 serious financial problems 22 serious physical disease – inability to work or daily activities  9 serious disease, accident or diagnosis for yourself or your family 23 serious injury - inability to work or daily activities  10 serious disease, accident or diagnosis for a close friend 24 pregnancy complications or miscarriage for yourself or your partner  11 death of a family member 25 victim of physical abuse or robbery  12 death of a friend 26 victim of sexual abuse  13 death of a loved pet 27 other as aversive experienced events (e.g. exams, car accident, domestic coal, earth quake,  14 divorce of the parents military combat) | | | | | | | | | | | | | | | | | | | | | | | | | | | |

**1.2. Recent adversity (RA)**

| **Supplementary Table 2.** The effect of recent adversity (RA; in the table referred as group) on autonomic (SCRs) and subjective (fear ratings) measures during fear acquisition, extinction and RI-induced ROF. Significant main effects of and interactions with adversity group are highlighted in blue font.   \|  \|  \| F \| df, df_error_ \| p \| Eta² \| \| --- \| --- \| --- \| --- \| --- \| --- \| \| **SCR** \|  \|  \|  \|  \|  \| \| Acquisition \| stimulus \| 50.57 \| 1/66 \| <0.001 \| 0.43 \| \|  \| group \| <1 \| 1/66 \| 0.599 \| <0.01 \| \|  \| stimulus*group \| <1 \| 1/66 \| 0.364 \| 0.01 \| \| 1^st^ half \| stimulus \| 12.55 \| 1/71 \| 0.001 \| 0.15 \| \| (fear recall) \| group \| 1.69 \| 1/71 \| 0.199 \| 0.02 \| \|  \| **stimulus*group** \| **4.43** \| **1/71** \| **0.039^1^** \| **0.06** \| \| 2^nd^ half \| stimulus \| 3.14 \| 1/70 \| 0.081 \| 0.04 \| \|  \| group \| <1 \| 1/70 \| 0.45 \| 0.01 \| \|  \| stimulus*group \| <1 \| 1/70 \| 0.339 \| 0.01 \| \| RI-test \| time \| 45.03 \| 1/69 \| <0.001 \| 0.40 \| \|  \| group \| 1.43 \| 1/69 \| 0.235 \| 0.02 \| \|  \| time*group \| <1 \| 1/69 \| 0.316 \| 0.02 \| \|  \| stimulus \| 3.99 \| 1/69 \| 0.05 \| 0.06 \| \|  \| stimulus*group \| 1.89 \| 1/69 \| 0.173 \| 0.03 \| \|  \| time*stimulus \| <1 \| 1/69 \| 0.498 \| 0.01 \| \|  \| **time*stimulus*group** \| **4.01** \| **1/69** \| **0.049^2^** \| **0.06** \| \| **Ratings** \|  \|  \|  \|  \|  \| \| Acquisition \| time \| 130.28 \| 1/64 \| < 0.001 \| 0.67 \| \|  \| group \| 3.07 \| 1/64 \| 0.084 \| 0.05 \| \|  \| time*group \| <1 \| 1/64 \| 0.760 \| <0.01 \| \|  \| stimulus \| 103.09 \| 1/64 \| <0.001 \| 0.62 \| \|  \| stimulus*group \| <1 \| 1/64 \| 0.795 \| <0.01 \| \|  \| time*stimulus \| 113.64 \| 1/64 \| < 0.001^3^ \| 0.64 \| \|  \| time*stimulus*group \| <1 \| 1/64 \| 0.840 \| <0.01 \| \| Extinction \| time \| 37.08 \| 1/66 \| <0.001 \| 0.36 \| \|  \| group \| 2.98 \| 1/66 \| 0.089 \| 0.04 \| \|  \| time*group \| 1.31 \| 1/66 \| 0.257 \| 0.02 \| \|  \| stimulus \| 92.98 \| 1/66 \| <0.001 \| 0.59 \| \|  \| stimulus*group \| <1 \| 1/66 \| 0.612 \| <0.01 \| \|  \| time*stimulus \| 49.98 \| 1/66 \| <0.001^4^ \| 0.43 \| \|  \| time*stimulus*group \| <1 \| 1/66 \| 0.997 \| <0.01 \| \| RI-test \| time \| 26.56 \| 1/64 \| <0.001 \| 0.29 \| \|  \| group \| 3.21 \| 1/64 \| 0.078 \| 0.05 \| \|  \| time*group \| 1.91 \| 1/64 \| 0.172 \| 0.03 \| \|  \| stimulus \| 35.43 \| 1/64 \| <0.001 \| 0.36 \| \|  \| stimulus*group \| 1.30 \| 1/64 \| 0.259 \| 0.02 \| \|  \| time*stimulus \| 4.18 \| 1/64 \| 0.045^5^ \| 0.06 \| \|  \| time*stimulus*group \| 1.38 \| 1/64 \| 0.245 \| 0.02 \|   **^1^** due to CS discrimination (CS+>CS-) within the RA-  **^2^** beside a general response increase RA- group shows a CS discrimination (CS+>CS-)  **^3^** due to higher ratings after acq in general and especially to CS+  **^4^** due to decreased responses after ext  **^5^** due to increased and differentiated (CS+>CS-) ratings after RI  **Supplementary Table 3.** The effect of dimensional measures ofRA (i.e. cumulative exposure to recent adversity) on autonomic (SCRs) and subjective (fear ratings) measures during fear acquisition, extinction and RI-induced ROF. Significant main effects of and interactions with adversity group are highlighted in blue font.   \|  \|  \| r \| p \| \| --- \| --- \| --- \| --- \| \| **SCR** \|  \|  \|  \| \| RA \| Acquisition \| -0.140 \| 0.229 \| \|  \| 1^st^ half Extinction \| -0.126 \| 0.286 \| \|  \| 2^nd^ half Extinction \| -0.004 \| 0.975 \| \|  \| **RI** \| **-0.281** \| **0.016** \| \| **Ratings** \|  \|  \|  \| \| RA \| Acquisition \| -0.082 \| 0.505 \| \|  \| before Extinction \| -0.111 \| 0.363 \| \|  \| after Extinction \| -0.124 \| 0.314 \| \|  \| RI \| 0.164 \| 0.189 \|   **Supplementary Table 4.** Neural activation of a whole brain analyses reflecting group differences between individuals exposed and not exposed to recent adversity (RA+ and RA- respectively) for acquisition, early extinction and late extinction. Clusters with a minimum size of k_min_=10^1^ were considered.   \| **acquisition** \| **brain area** \| **x** \| **y** \| **z** \| **T** \| **p(uc)** \| **p(svc_FWE_)** \| \| --- \| --- \| --- \| --- \| --- \| --- \| --- \| --- \| \| RA-_Acq(CS+>CS-)_ \| med. Temp. gyrus(L) \| -60 \| -10 \| -12 \| 3.67 \| <0.001 \| - \| \| >RA+_Acq(CS+>CS-)_ \|  \| -56 \| -54 \| 8 \| 3.43 \| <0.001 \| - \| \| RA-_+Acq(CS+>CS-)_ <RA+_Acq(CS+>CS-)_ \| none \|  \|  \|  \|  \|  \|  \| \| **early extinction** \| **brain area** \| **x** \| **y** \| **z** \| **T** \| **p(uc)** \| **p(svc_FWE_)** \| \| RA-_Ext1(CS+>CS-)_ \| lingual gyrus(R) \| 14 \| -34 \| -10 \| 3.71 \| <0.001 \| - \| \| > RA+_Ext1(CS+>CS-)_ \| hippocampus(R) \| 24 \| -36 \| -4 \| 3.64 \| <0.001 \| 0.035 \| \|  \| WM \| 22 \| -34 \| -6 \| 3.65 \| <0.001 \| - \| \|  \|  \| -36 \| -18 \| 4 \| 3.51 \| <0.001 \| - \| \| RA-_Ext1(CS+>CS-)_ \| cerebellum \| 0 \| -66 \| -2 \| 4.24 \| <0.001 \| - \| \| < RA+_Ext1(CS+>CS-)_ \| precentral gyrus(R) \| 40 \| 4 \| 32 \| 4.17 \| <0.001 \| - \| \|  \| cerebellum(R) \| 22 \| -68 \| -42 \| 4.00 \| <0.001 \| - \| \|  \| caudate(R) \| 14 \| 10 \| -10 \| 3.99 \| <0.001 \| - \| \|  \| med. occipital lobe(L) \| -36 \| -94 \| 2 \| 3.73 \| <0.001 \| - \| \|  \| med. frontal gyrus(R) \| 40 \| 34 \| 30 \| 3.64 \| <0.001 \| - \| \| **late extinction** \| **brain area** \| **x** \| **y** \| **z** \| **T** \| **p(uc)** \| **p(svc_FWE_)** \| \| RA-_Ext2(CS+>CS)_ \| none \|  \|  \|  \|  \|  \|  \| \| >RA+_Ext2(CS+>CS-)_ \|  \|  \|  \|  \|  \|  \|  \| \| RA-_Ext2(CS+>CS-)_ <RA+_Ext2(CS+>CS-)_ \| WM \| -36 \| -6 \| -16 \| 3.94 \| <0.001 \| - \|   1 = an exception was made for ROIs  Ext1 = first half of extinction – trial 1-7  Ext2 = second half of extinction – trial 8 - 14  R = right; L = left  WM = white matter   \| **Supplementary Table 5.** Neural activation of a whole brain analyses at p<0.001uc reflecting group differences in CS discrimination (CS+>CS-) in RI for individuals exposed (RA+) and unexposed (RA-) to recent adversity with a k_min_=10^1^. \| \| \| \| \| \| \| \| \| --- \| --- \| --- \| --- \| --- \| --- \| --- \| --- \| \| Contrast \| brain area \| x \| y \| z \| T \| p(uc) \| p(svc_FWE_) \| \| RA-_RI(CS+>CS-)_>RA+ \| thalamus(L) \| -2 \| -14 \| 14 \| 4.30 \| <0.001 \| 0.010 \| \| _RI(CS+>CS-)_ \| hippocampus(L) \| -28 \| -14 \| -24 \| 3.76 \| <0.001 \| 0.026 \| \|  \| hippocampus(R) \| 22 \| -12 \| -16 \| 3.34 \| <0.001 \| 0.080 \| \|  \| amygdala(R) \| 20 \| -10 \| -14 \| 3.39 \| <0.001 \| 0.038 \| \|  \| inf. parietal gyrus(L) \| -28 \| -74 \| 42 \| 5.49 \| <0.001 \| 0.030^+^ \| \|  \| postcentral gyrus(R) \| 22 \| -42 \| 58 \| 5.34 \| <0.001 \| 0.049^+^ \| \|  \| precentral gyrus(R) \| 24 \| -26 \| 52 \| 4.50 \| <0.001 \| 0.021^+^ \| \|  \| sup. frontal gyrus(L) \| -22 \| 44 \| 40 \| 4.66 \| <0.001 \| - \| \|  \| precuneus(L) \| -2 \| -72 \| 34 \| 4.56 \| <0.001 \| - \| \|  \|  \| -10 \| -64 \| 48 \| 3.93 \| <0.001 \| - \| \|  \| med. temporal gyrus(R) \| 58 \| -42 \| 8 \| 4.46 \| <0.001 \| - \| \|  \| insula(R) \| 34 \| -14 \| 20 \| 4.45 \| <0.001 \| - \| \|  \| sup. temporal gyrus(L) \| -46 \| -40 \| 12 \| 4.34 \| <0.001 \| - \| \|  \| cerebellar vermis \| 0 \| -42 \| 2 \| 4.31 \| <0.001 \| - \| \|  \|  \| 0 \| -64 \| 6 \| 4.27 \| <0.001 \| - \| \|  \| sup. temporal gyrus(R) \| 68 \| -14 \| 2 \| 4.23 \| <0.001 \| - \| \|  \|  \| 56 \| -14 \| 6 \| 4.04 \| <0.001 \| - \| \|  \| med. frontal gyrus(R) \| 28 \| 40 \| 46 \| 4.16 \| <0.001 \| - \| \|  \| sup. frontal gyrus(R) \| 16 \| 62 \| 20 \| 4.14 \| <0.001 \| - \| \|  \| angular gyrus(R) \| 36 \| -66 \| 48 \| 4.14 \| <0.001 \| - \| \|  \| cerebellum(L) \| -4 \| -82 \| -22 \| 4.10 \| <0.001 \| - \| \|  \| med. frontal gyrus(L) \| -48 \| 14 \| 38 \| 4.02 \| <0.001 \| - \| \|  \| sup. occipital gyrus(R) \| 24 \| -66 \| 34 \| 4.00 \| <0.001 \| - \| \|  \| med. sup. frontal gyrus(L) \| -4 \| 64 \| 20 \| 4.00 \| <0.001 \| - \| \|  \| cerebellum(R) \| 16 \| -82 \| -28 \| 3.99 \| <0.001 \| - \| \|  \|  \| 32 \| -84 \| -34 \| 3.75 \| <0.001 \| - \| \|  \| inf.orbitofrontal gyrus(L) \| -40 \| 42 \| -10 \| 3.93 \| <0.001 \| - \| \|  \| inf. temporal gyrus(L) \| -60 \| -16 \| -26 \| 3.91 \| <0.001 \| - \| \|  \| supp. motor area(R) \| 6 \| -14 \| 52 \| 3.84 \| <0.001 \| - \| \|  \| fusiform gyrus(R) \| 40 \| -38 \| -24 \| 3.83 \| <0.001 \| - \| \|  \| fusiform gyrus(L) \| -28 \| -14 \| -24 \| 3.76 \| <0.001 \| - \| \|  \| caudate(R) \| 18 \| 12 \| 20 \| 3.75 \| <0.001 \| - \| \|  \| inf. parietal lobe(L) \| -50 \| -46 \| 48 \| 3.50 \| <0.001 \| - \| \|  \| WM \| 24 \| -26 \| 52 \| 5.58 \| <0.001 \| - \| \|  \|  \| -16 \| 52 \| -8 \| 4.89 \| <0.001 \| - \| \|  \|  \| -22 \| -32 \| 12 \| 4.50 \| <0.001 \| - \| \|  \|  \| 0 \| -42 \| 2 \| 4.31 \| <0.001 \| - \| \|  \|  \| -14 \| -30 \| 60 \| 4.08 \| <0.001 \| - \| \|  \|  \| -16 \| -18 \| 62 \| 3.91 \| <0.001 \| - \| \| RA-_RI(CS+>CS-)_<RA+ _RI(CS+>CS-)_ \| none \|  \|  \|  \|  \|  \|  \|   1 = except for ROI-based analyses  + = whole brain FWE-corrected (non-ROI-based analyses)  **1.3. Childhood adversity (CA)**  **Supplementary Table 6.** The effect of childhood adversity (CA; in the table referred to as group 2) on autonomic (SCRs) and subjective (fear ratings) measures during fear acquisition, extinction and RI-induced ROF. Significant main effects of and interactions with adversity group are highlighted in blue font.   \|  \|  \| F \| df, df_error_ \| p \| Eta² \| \| --- \| --- \| --- \| --- \| --- \| --- \| \| **SCR** \|  \|  \|  \|  \|  \| \| Acquisition \| stimulus \| 47.93 \| 1/66 \| <0.001 \| 0.42 \| \|  \| group \| <1 \| 1/66 \| 0.865 \| <0.01 \| \|  \| stimulus*group \| <1 \| 1/66 \| 0.846 \| <0.01 \| \| 1^st^ half Extinction \| stimulus \| 9.96 \| 1/71 \| 0.002 \| 0.12 \| \| (fear recall) \| group \| 3.30 \| 1/71 \| 0.074 \| 0.04 \| \|  \| stimulus*group \| 1.56 \| 1/71 \| 0.215 \| 0.02 \| \| 2^nd^ half \| stimulus \| 3.40 \| 1/70 \| 0.070 \| 0.05 \| \| Extinction \| group \| <1 \| 1/70 \| 0.675 \| <0.01 \| \|  \| stimulus*group \| <1 \| 1/70 \| 0.969 \| <0.01 \| \| RI-test \| time \| 43.27 \| 1/69 \| <0.001 \| 0.39 \| \|  \| group \| <1 \| 1/69 \| 0.991 \| <0.01 \| \|  \| time*group \| <1 \| 1/69 \| 0.835 \| <0.01 \| \|  \| stimulus \| 3.64 \| 1/69 \| 0.061 \| 0.05 \| \|  \| stimulus*group \| <1 \| 1/69 \| 0.669 \| <0.01 \| \|  \| time*stimulus \| <1 \| 1/69 \| 0.645 \| <0.01 \| \|  \| time*stimulus*group \| <1 \| 1/69 \| 0.357 \| 0.01 \| \| **Ratings** \|  \|  \|  \|  \|  \| \| Acquisition \| time \| 130.36 \| 1/64 \| <0.001 \| 0.67 \| \|  \| group \| 1.08 \| 1/64 \| 0.302 \| 0.02 \| \|  \| time*group \| <1 \| 1/64 \| 0.861 \| <0.01 \| \|  \| stimulus \| 104.29 \| 1/64 \| <0.001 \| 0.62 \| \|  \| stimulus*group \| <1 \| 1/64 \| 0.528 \| 0.01 \| \|  \| time*stimulus \| 119.32 \| 1/64 \| <0.001^6^ \| 0.65 \| \|  \| time*stimulus*group \| 3.70 \| 1/64 \| 0.059 \| 0.06 \| \| Extinction \| time \| 39.22 \| 1/66 \| <0.001 \| 0.37 \| \|  \| group \| <1 \| 1/66 \| 0.409 \| 0.01 \| \|  \| time*group \| <1 \| 1/66 \| 0.398 \| 0.01 \| \|  \| stimulus \| 95.02 \| 1/66 \| <0.001 \| 0.60 \| \|  \| stimulus*group \| <1 \| 1/66 \| 0.978 \| <0.01 \| \|  \| time*stimulus \| 51.15 \| 1/66 \| <0.001^7^ \| 0.44 \| \|  \| time*stimulus*group \| <1 \| 1/66 \| 0.557 \| 0.01 \| \| RI-test \| time \| 29.10 \| 1/64 \| <0.001 \| 0.31 \| \|  \| group \| <1 \| 1/64 \| 0.964 \| <0.01 \| \|  \| time*group \| 1.81 \| 1/64 \| 0.183 \| 0.03 \| \|  \| stimulus \| 36.89 \| 1/64 \| <0.001 \| 0.37 \| \|  \| stimulus*group \| <1 \| 1/64 \| 0.952 \| <0.01 \| \|  \| time*stimulus \| 4.84 \| 1/64 \| 0.031^8^ \| 0.07 \| \|  \| time*stimulus*group \| <1 \| 1/64 \| 0.600 \| <0.01 \|   **^6^** due to higher ratings after acquisition particularly to the CS+  **^7^** due to decreased and less differential ratings after extinction  **^8^** due to increased and differential (CS+>CS-) ratings after RI  **Supplementary Table 7.** The effect of dimensional measures of CA (i.e. cumulative exposure to childhood adversity) on autonomic (SCRs) and subjective (fear ratings) measures during fear acquisition, extinction and RI-induced ROF. Significant main effects of and interactions with adversity group are highlighted in blue font.   \|  \|  \| r \| p \| \| --- \| --- \| --- \| --- \| \| **SCR** \|  \|  \|  \| \| CA \| Acquisition \| -0.141 \| 0.224 \| \|  \| 1^st^ half Extinction \| -0.139 \| 0.239 \| \|  \| 2^nd^ half Extinction \| -0.036 \| 0.760 \| \|  \| **RI** \| **-0.268** \| **0.022** \| \| **Ratings** \|  \|  \|  \| \| CA \| Acquisition \| -0.021 \| 0.865 \| \|  \| before Extinction \| 0.015 \| 0.901 \| \|  \| after Extinction \| -0.050 \| 0.683 \| \|  \| RI \| -0.001 \| 0.991 \|   **Supplementary Table 8.** Neural activation reflecting group differences for acquisition, early extinction and late extinction for CA. Clusters with a minimum of k_min_=10^1^ were considered.   \| **acquisition** \| **brain area** \| **x** \| **y** \| **z** \| **T** \| **p(uc)** \| **p(svc_FWE_)** \| \| --- \| --- \| --- \| --- \| --- \| --- \| --- \| --- \| \| CA-_Acq(CS+>CS-)_ \| lingual gyrus(R) \| 12 \| -30 \| -10 \| 4.25 \| <0.001 \| - \| \| >CA+_Acq(CS+>CS-)_ \| Precentral gyrus(L) \| -32 \| -10 \| 64 \| 3.44 \| <0.001 \| - \| \| CA-_+Acq(CS+>CS-)_ \| none \|  \|  \|  \|  \|  \|  \| \| <CA+_Acq(CS+>CS-)_ \|  \|  \|  \|  \|  \|  \|  \| \| **early extinction** \| **brain area** \| **x** \| **y** \| **z** \| **T** \| **p(uc)** \| **p(svc_FWE_)** \| \| CA-_Ext1(CS+>CS-)_ \| calcarine fissure(R) \| 12 \| -86 \| 6 \| 4.18 \| <0.001 \| - \| \| > CA+_Ext1(CS+>CS-)_ \| temporal operculum(R) \| 44 \| -26 \| 20 \| 3.89 \| <0.001 \| - \| \|  \| insula cortex(L) \| -34 \| -22 \| 12 \| 3.75 \| <0.001 \| - \| \|  \| med. temporal gyrus(L) \| -50 \| 4 \| -24 \| 3.62 \| <0.001 \| - \| \|  \| Rectal gyrus(L) \| -4 \| 34 \| -20 \| 3.44 \| <0.001 \| - \| \| CA-_Ext1(CS+>CS-)_ \| none \|  \|  \|  \|  \|  \|  \| \| < CA+_Ext1(CS+>CS-)_ \|  \|  \|  \|  \|  \|  \|  \| \| **late extinction** \| **brain area** \| **x** \| **y** \| **z** \| **T** \| **p(uc)** \| **p(svc_FWE_)** \| \| CA-_Ext2(CS+>CS-)_ \| none \|  \|  \|  \|  \|  \|  \| \| >CA+_Ext2(CS+>CS-)_ \|  \|  \|  \|  \|  \|  \|  \| \| CA-_Ext2(CS+>CS-)_ \| WM \| -44 \| -48 \| 14 \| 4.00 \| <0.001 \| - \| \| <CA+_Ext2(CS+>CS-)_ \|  \| -20 \| -34 \| -2 \| 3.95 \| <0.001 \| - \| \| **RI** \| **brain area** \| **x** \| **y** \| **z** \| **T** \| **p(uc)** \| **p(svc_FWE_)** \| \| CA-_RI(CS+>CS-)_  >CA+_RI(CS+>CS-)_ \| precentral gyrus(L) \| -32 \| 8 \| 38 \| 4.09 \| <0.001 \| - \| \| CA-_RI(CS+>CS-)_ \| sup. frontal gyrus(R) \| 14 \| 42 \| 28 \| 5.07 \| <0.001 \| - \| \| <CA+ _RI(CS+>CS-)_ \|  \| 22 \| 2 \| 60 \| 4.12 \| <0.001 \| - \| \|  \| calcarine fissure(R) \| 18 \| -78 \| 14 \| 4.66 \| <0.001 \| - \| \|  \| cerebellum(R) \| 30 \| -56 \| -28 \| 3.64 \| <0.001 \| - \| \|  \| WM \| -16 \| -32 \| 52 \| 4.24 \| <0.001 \| - \| \|  \|  \| 8 \| -50 \| -26 \| 3.89 \| <0.001 \| - \| \|  \|  \| 22 \| 36 \| 14 \| 3.56 \| <0.001 \| - \| \|  \|  \| -10 \| -26 \| -6 \| 3.55 \| <0.001 \| - \|   1 = except for ROI-based analyses  **1.3. Allostatic load (AL)**  **Supplementary Table 9.** The effect of allostatic load (AL) (i.e. cumulative exposure to adversity throughout life) on autonomic (SCRs) and subjective (fear ratings) measures during fear acquisition, extinction and RI-induced ROF. Significant main effects of and interactions with adversity group are highlighted in blue font.   \|  \|  \| r \| p \| \| --- \| --- \| --- \| --- \| \| **SCR** \|  \|  \|  \| \| AL \| Acquisition \| -0.061 \| 0.602 \| \|  \| 1^st^ half Extinction \| -0.186 \| 0.116 \| \|  \| 2^nd^ half Extinction \| 0.042 \| 0.723 \| \|  \| **RI** \| **-0.238** \| **0.043** \| \| **Ratings** \|  \|  \|  \| \| AL \| Acquisition \| 0.013 \| 0.916 \| \|  \| before Extinction \| 0.002 \| 0.987 \| \|  \| after Extinction \| -0.155 \| 0.206 \| \|  \| RI \| 0.120 \| 0.337 \|  \| **Supplementary Table 10.** Neural activation reflecting group differences for acquisition, early extinction and late extinction for allostatic load (AL) (overall accumulation of stressful life events throughout life) derived from regression analyses. Clusters with a minimum size of k_min_=10^1^ were considered. \| \| \| \| \| \| \| \| \| --- \| --- \| --- \| --- \| --- \| --- \| --- \| --- \| \| **acquisition** \| **brain area** \| **x** \| **y** \| **z** \| **T** \| **p(uc)** \| **p(svc_FWE_)** \| \| pos. ass. with AL \| med. temporal gyrus (R) \| 48 \| -44 \| -4 \| 4.7 \| <0.001 \| - \| \|  \| inf. frontal operculum (R) \| 50 \| 12 \| 36 \| 4.19 \| <0.001 \| - \| \|  \| med. frontal gyrus (R) \| 44 \| 56 \| 8 \| 3.84 \| <0.001 \| - \| \|  \| cerebellum (R) \| 22 \| -76 \| -20 \| 3.62 \| <0.001 \| - \| \|  \| WM \| 40 \| -66 \| -2 \| 4.66 \| <0.001 \| - \| \|  \|  \|  \|  \|  \|  \|  \|  \| \|  \|  \| -16 \| -38 \| 8 \| 4.01 \| <0.001 \| - \| \| neg. ass. with AL \| sup. temporal gyrus (L) \| -44 \| -4 \| -14 \| 3.99 \| <0.001 \| - \| \| **early extinction** \| **brain area** \| **x** \| **y** \| **z** \| **T** \| **p(uc)** \| **p(svc_FWE_)** \| \| pos. ass. with AL \| med. cingulate gyrus (R) \| 12 \| 20 \| 40 \| 3.85 \| <0.001 \| - \| \| neg. ass. with AL \| amygdala (R) \| 20 \| -2 \| -26 \| 3.35 \| 0.001 \| 0.041 \| \|  \| postcentral gyrus (R) \| 58 \| -2 \| 24 \| 3.40 \| 0.001 \| - \| \|  \| WM \| 0 \| 4 \| 22 \| 4.36 \| <0.001 \| - \| \| **late extinction** \| **brain area** \| **x** \| **y** \| **z** \| **T** \| **p(uc)** \| **p(svc_FWE_)** \| \| pos. ass. with AL \| WM \| -14 \| 34 \| -10 \| 4.47 \| <0.001 \| - \| \| neg. ass. with AL \| WM \| -30 \| -2 \| 20 \| 3.69 \| <0.001 \| - \| \| **RI** \| **brain area** \| **x** \| **y** \| **z** \| **T** \| **p(uc)** \| **p(svc_FWE_)** \| \| pos. ass. with AL \| cerebellum (R) \| 4 \| -56 \| -30 \| 3.79 \| <0.001 \| - \| \|  \|  \| 18 \| -58 \| -44 \| 3.72 \| <0.001 \| - \| \|  \| supp. Motor area (R) \| 14 \| 12 \| 62 \| 3.68 \| <0.001 \| - \| \| neg. ass. with AL \| thalamus (L) \| -4 \| -10 \| 6 \| 4.38 \| <0.001 \| 0.008 \| \|  \| thalamus (R) \| 6 \| -18 \| 14 \| 3.76 \| <0.001 \| 0.048 \| \|  \| ACC (L) \| -8 \| 30 \| 22 \| 4.29 \| <0.001 \| 0.006 \| \|  \| ACC (R) \| 6 \| 36 \| 22 \| 3.80 \| <0.001 \| 0.034 \| \|  \| hippocampus (R) \| 22 \| -38 \| 4 \| 3.35 \| <0.001 \| 0.080 \| \|  \| sup. temporal gyrus (R) \| 68 \| -14 \| 6 \| 7.02 \| <0.001 \| <0.001^+^ \| \|  \| cerebellum (R) \| 18 \| -78 \| -24 \| 6.59 \| <0.001 \| 0.001^+^ \| \|  \|  \| 0 \| -42 \| 2 \| 4.02 \| <0.001 \| - \| \|  \| med. sup. frontal gyrus (R) \| 12 \| 64 \| 20 \| 6.11 \| <0.001 \| 0.004^+^ \| \|  \| angular gyrus (L) \| -38 \| -68 \| 46 \| 5.47 \| <0.001 \| 0.034^+^ \| \|  \| lingual gyrus (L) \| -28 \| -60 \| -2 \| 5.12 \| <0.001 \| - \| \|  \|  \| -14 \| -72 \| -2 \| 4.14 \| <0.001 \| - \| \|  \| angular gyrus (R) \| 38 \| -58 \| 44 \| 4.83 \| <0.001 \| - \| \|  \|  \| 44 \| -50 \| 28 \| 4.03 \| <0.001 \| - \| \|  \| precuneus (L) \| -8 \| -62 \| 38 \| 4.67 \| <0.001 \| - \| \|  \| cerebellum (L) \| -44 \| -72 \| -46 \| 4.51 \| <0.001 \| - \| \|  \|  \| -10 \| -82 \| -28 \| 4.44 \| <0.001 \| - \| \|  \| med. temporal gyrus (L) \| -64 \| -40 \| 6 \| 4.42 \| <0.001 \| - \| \|  \|  \| -58 \| -48 \| -12 \| 4.33 \| <0.001 \| - \| \|  \| inf. temporal gyrus (R) \| 54 \| -58 \| -10 \| 4.30 \| <0.001 \| - \| \|  \| postcentral gyrus (L) \| -62 \| -12 \| 14 \| 4.14 \| <0.001 \| - \| \|  \| inf. frontal operculum (R) \| 54 \| 8 \| 14 \| 4.11 \| <0.001 \| - \| \|  \| lingual gyrus (R) \| 8 \| -88 \| -6 \| 4.03 \| <0.001 \| - \| \|  \|  \| 20 \| -74 \| 2 \| 3.70 \| <0.001 \| - \| \|  \| caudate (R) \| 10 \| 12 \| 4 \| 4.02 \| <0.001 \| - \| \|  \|  \| 16 \| 8 \| 18 \| 3.96 \| <0.001 \| - \| \|  \| med. temporal gyrus (R) \| 48 \| -48 \| 12 \| 3.99 \| <0.001 \| - \| \|  \| inf. frontal gyrus (L) \| -52 \| 38 \| 8 \| 3.80 \| <0.001 \| - \| \|  \| calcarine fissure (L) \| -2 \| -92 \| -8 \| 3.79 \| <0.001 \| - \| \|  \| rolandic operculum (L) \| -44 \| -10 \| 16 \| 3.76 \| <0.001 \| - \| \|  \| med. orbitofrontal gyrus (L) \| -38 \| 50 \| -10 \| 3.75 \| <0.001 \| - \| \|  \| fusiform gyrus (L) \| -38 \| -60 \| -12 \| 3.64 \| <0.001 \| - \| \|  \| WM \| 26 \| -28 \| 50 \| 4.67 \| <0.001 \| - \| \|  \|  \| -40 \| -44 \| 4 \| 4.34 \| <0.001 \| - \| \|  \|  \| -12 \| -46 \| 24 \| 4.29 \| <0.001 \| - \| \|  \|  \| 32 \| -10 \| 18 \| 4.24 \| <0.001 \| - \| \|  \|  \| -4 \| 14 \| -28 \| 4.14 \| <0.001 \| - \| \|  \|  \| -8 \| -20 \| -24 \| 4.12 \| <0.001 \| - \| \|  \|  \| -44 \| -50 \| 16 \| 4.08 \| <0.001 \| - \| \|  \|  \| -24 \| -70 \| 18 \| 4.07 \| <0.001 \| - \| \|  \|  \| -32 \| -58 \| 20 \| 3.67 \| <0.001 \| - \| \|  \|  \| -26 \| -34 \| 6 \| 3.58 \| <0.001 \| - \| \| 1 = except for ROI-based analyses  pos. ass. = positive association  neg. ass. = negative association  WM = white matter  ^+^ = whole brain FWE-corrected \|  \|  \|  \|  \|  \|  \|  \|   **1.4. Mismatch groups**  Exploratory investigations of all four subgroups (mismatch hypothesis), differing in presence and absence of early and recent adversity (CA+/RA+, CA+/RA-, CA-/RA+, CA-/RA-), revealed a main effect of group on SCRs during fear recall only [F(3,69)=2.76, p=0.048, η²=0.11, **Supplementary Table 11**] driven by the lowest risk group (CA-/RA-) showing globally higher SCRs than any other group. While no interaction with group on SCRs was observed during fear acquisition and extinction, a significant CS type*time*group interaction was observed during RI-test [F(4,67)=4.41, p=0.007, η²=0.17]. Thereby, only the allostatic load group (RA+/CA+) shows a significant time*cs interaction [F(1,20)=7.31, p=0.014, η²=0.27], driven by a significant increase of the CS- (t=4.88, p<0.001), but not the CS+ (t=1.58, p=0.129) from extinction to RI-test (**Supplementary Table 11**).  For fear ratings a main effect of group is observed during acquisition [F(3,62)=3.042, p=0.035, η²=0.13] that is driven by the CA-/RA+ (mismatch) group that shows higher ratings than the other three groups (**Supplementary Table 11**).  Contrasting the allostatic load group on a neuro-functional level against all other groups for discrimination (CS+>CS-) revealed no significant activation differences in any of our ROIs (**Supplementary Table 12** shows also explorative analyses at p<0.001).  **Supplementary Table 11.** The effect of environmental mismatch between recent and childhood adversity on autonomic (SCRs) and subjective (fear ratings) measures during fear acquisition, extinction and RI-induced ROF. Significant main effects of and interactions with adversity group are highlighted in blue font.   \|  \|  \| F \| df, df_error_ \| p \| Eta² \| \| --- \| --- \| --- \| --- \| --- \| --- \| \| **SCR** \|  \|  \|  \|  \|  \| \| Acquisition \| stimulus \| 50.32 \| 1/64 \| <0.001 \| 0.44 \| \|  \| group \| <1 \| 3/64 \| 0.900 \| 0.01 \| \|  \| stimulus*group \| <1 \| 3/64 \| 0.510 \| 0.04 \| \| 1^st^ half Extinction \| stimulus \| 11.24 \| 1/69 \| 0.001 \| 0.14 \| \| (fear recall) \| **group** \| **2.76** \| **3/69** \| **0.048^10^** \| **0.11** \| \|  \| stimulus*group \| 1.75 \| 3/69 \| 0.165 \| 0.07 \| \| 2^nd^half Extinction \| stimulus \| 2.01 \| 1/68 \| 0.160 \| 0.03 \| \|  \| group \| 1.86 \| 1/68 \| 0.145 \| 0.08 \| \|  \| stimulus*group \| 1.24 \| 3/68 \| 0.303 \| 0.05 \| \| RI-test \| time \| 43.03 \| 1/67 \| <0.001 \| 0.39 \| \|  \| group \| 1.34 \| 3/67 \| 0.269 \| 0.06 \| \|  \| time*group \| <1 \| 1/67 \| 0.750 \| 0.02 \| \|  \| stimulus \| 4.52 \| 1/67 \| 0.037 \| 0.06 \| \|  \| stimulus*group \| <1 \| 3/67 \| 0.430 \| 0.04 \| \|  \| time*stimulus \| 1.49 \| 1/67 \| 0.226 \| 0.02 \| \|  \| **time*stimulus*group** \| **4.41** \| **3/67** \| **0.007^11^** \| **0.17** \| \| **Ratings** \|  \|  \|  \|  \|  \| \| Acquisition \| time \| 125.36 \| 1/62 \| <0.001 \| 0.67 \| \|  \| **group** \| **3.04** \| **3/62** \| **0.035^12^** \| **0.13** \| \|  \| time*group \| <1 \| 3/62 \| 0.982 \| <0.01 \| \|  \| stimulus \| 102.85 \| 1/62 \| <0.001 \| 0.62 \| \|  \| stimulus*group \| <1 \| 3/62 \| 0.744 \| 0.02 \| \|  \| time*stimulus \| 118.38 \| 1/62 \| <0.001^13^ \| 0.66 \| \|  \| time*stimulus*group \| 1.66 \| 3/62 \| 0.185 \| 0.07 \| \| Extinction \| time \| 41.05 \| 1/64 \| <0.001 \| 0.39 \| \|  \| group \| 1.36 \| 3/64 \| 0.263 \| 0.06 \| \|  \| time*group \| 2.02 \| 3/64 \| 0.120 \| 0.09 \| \|  \| Stimulus \| 90.85 \| 1/64 \| <0.001 \| 0.59 \| \|  \| stimulus*group \| <1 \| 3/64 \| 0.885 \| 0.01 \| \|  \| time*stimulus \| 51.44 \| 1/64 \| <0.001^14^ \| 0.45 \| \|  \| time*stimulus*group \| <1 \| 3/64 \| 0.591 \| 0.03 \| \| RI-test \| time \| 30.33 \| 1/62 \| <0.001 \| 0.33 \| \|  \| group \| 1.19 \| 1/62 \| 0.320 \| 0.06 \| \|  \| time*group \| 2.25 \| 3/62 \| 0.091 \| 0.10 \| \|  \| Stimulus \| 34.30 \| 1/62 \| <0.001 \| 0.36 \| \|  \| stimulus*group \| <1 \| 3/62 \| 0.702 \| 0.02 \| \|  \| time*stimulus \| 4.79 \| 1/62 \| 0.032^15^ \| 0.07 \| \|  \| time*stimulus*group \| <1 \| 3/62 \| 0.42 \| 0.04 \|   **^10^** group CA-/RA- shows higher responses as the other groups  **^11^** mainly driven by group CA+/RA+ that significantly differs from all other groups  **^12^** driven by group CA-/RA+ that significantly differs from group CA-/RA- (p=0.007) and CA+/RA+ (p=0.023) and on a trend level also  from group CA+/RA- (p=0.057)  **^13^** due to higher ratings after acq in general and especially to CS+  ^14^ due to decreased and less differential ratings after ext  ^15^ due to increased and differentiated (CS+>CS-) ratings after RI |
| --- | --- | --- | --- | --- | --- | --- | --- | --- | --- | --- | --- | --- | --- | --- | --- | --- | --- | --- | --- | --- | --- | --- | --- | --- | --- | --- | --- | --- | --- | --- | --- | --- | --- | --- | --- | --- | --- | --- | --- | --- | --- | --- | --- | --- | --- | --- | --- | --- | --- | --- | --- | --- | --- | --- | --- | --- | --- | --- | --- | --- | --- | --- | --- | --- | --- | --- | --- | --- | --- | --- | --- | --- | --- | --- | --- | --- | --- | --- | --- | --- | --- | --- | --- | --- | --- | --- | --- | --- | --- | --- | --- | --- | --- | --- | --- | --- | --- | --- | --- | --- | --- | --- | --- | --- | --- | --- | --- | --- | --- | --- | --- | --- | --- | --- | --- | --- | --- | --- | --- | --- | --- | --- | --- | --- | --- | --- | --- | --- | --- | --- | --- | --- | --- | --- | --- | --- | --- | --- | --- | --- | --- | --- | --- | --- | --- | --- | --- | --- | --- | --- | --- | --- | --- | --- | --- | --- | --- | --- | --- | --- | --- | --- | --- | --- | --- | --- | --- | --- | --- | --- | --- | --- | --- | --- | --- | --- | --- | --- | --- | --- | --- | --- | --- | --- | --- | --- | --- | --- | --- | --- | --- | --- | --- | --- | --- | --- | --- | --- | --- | --- | --- | --- | --- | --- | --- | --- | --- | --- | --- | --- | --- | --- | --- | --- | --- | --- | --- | --- | --- | --- | --- | --- | --- | --- | --- | --- | --- | --- | --- | --- | --- | --- | --- | --- | --- | --- | --- | --- | --- | --- | --- | --- | --- | --- | --- | --- | --- | --- | --- | --- | --- | --- | --- | --- | --- | --- | --- | --- | --- | --- | --- | --- | --- | --- | --- | --- | --- | --- | --- | --- | --- | --- | --- | --- | --- | --- | --- | --- | --- | --- | --- | --- | --- | --- | --- | --- | --- | --- | --- | --- | --- | --- | --- | --- | --- | --- | --- | --- | --- | --- | --- | --- | --- | --- | --- | --- | --- | --- | --- | --- | --- | --- | --- | --- | --- | --- | --- | --- | --- | --- | --- | --- | --- | --- | --- | --- | --- | --- | --- | --- | --- | --- | --- | --- | --- | --- | --- | --- | --- | --- | --- | --- | --- | --- | --- | --- | --- | --- | --- | --- | --- | --- | --- | --- | --- | --- | --- | --- | --- | --- | --- | --- | --- | --- | --- | --- | --- | --- | --- | --- | --- | --- | --- | --- | --- | --- | --- | --- | --- | --- | --- | --- | --- | --- | --- | --- | --- | --- | --- | --- | --- | --- | --- | --- | --- | --- | --- | --- | --- | --- | --- | --- | --- | --- | --- | --- | --- | --- | --- | --- | --- | --- | --- | --- | --- | --- | --- | --- | --- | --- | --- | --- | --- | --- | --- | --- | --- | --- | --- | --- | --- | --- | --- | --- | --- | --- | --- | --- | --- | --- | --- | --- | --- | --- | --- | --- | --- | --- | --- | --- | --- | --- | --- | --- | --- | --- | --- | --- | --- | --- | --- | --- | --- | --- | --- | --- | --- | --- | --- | --- | --- | --- | --- | --- | --- | --- | --- | --- | --- | --- | --- | --- | --- | --- | --- | --- | --- | --- | --- | --- | --- | --- | --- | --- | --- | --- | --- | --- | --- | --- | --- | --- | --- | --- | --- | --- | --- | --- | --- | --- | --- | --- | --- | --- | --- | --- | --- | --- | --- | --- | --- | --- | --- | --- | --- | --- | --- | --- | --- | --- | --- | --- | --- | --- | --- | --- | --- | --- | --- | --- | --- | --- | --- | --- | --- | --- | --- | --- | --- | --- | --- | --- | --- | --- | --- | --- | --- | --- | --- | --- | --- | --- | --- | --- | --- | --- | --- | --- | --- | --- | --- | --- | --- | --- | --- | --- | --- | --- | --- | --- | --- | --- | --- | --- | --- | --- | --- | --- | --- | --- | --- | --- | --- | --- | --- | --- | --- | --- | --- | --- | --- | --- | --- | --- | --- | --- | --- | --- | --- | --- | --- | --- | --- | --- | --- | --- | --- | --- | --- | --- | --- | --- | --- | --- | --- | --- | --- | --- | --- | --- | --- | --- | --- | --- | --- | --- | --- | --- | --- | --- | --- | --- | --- | --- | --- | --- | --- | --- | --- | --- | --- | --- | --- | --- | --- | --- | --- | --- | --- | --- | --- | --- | --- | --- | --- | --- | --- | --- | --- | --- | --- | --- | --- | --- | --- | --- | --- | --- | --- | --- | --- | --- | --- | --- | --- | --- | --- | --- | --- | --- | --- | --- | --- | --- | --- | --- | --- | --- | --- | --- | --- | --- | --- | --- | --- | --- | --- | --- | --- | --- | --- | --- | --- | --- | --- | --- | --- | --- | --- | --- | --- | --- | --- | --- | --- | --- | --- | --- | --- | --- | --- | --- | --- | --- | --- | --- | --- | --- | --- | --- | --- | --- | --- | --- | --- | --- | --- | --- | --- | --- | --- | --- | --- | --- | --- | --- | --- | --- | --- | --- | --- | --- | --- | --- | --- | --- | --- | --- | --- | --- | --- | --- | --- | --- | --- | --- | --- | --- | --- | --- | --- | --- | --- | --- | --- | --- | --- | --- | --- | --- | --- | --- | --- | --- | --- | --- | --- | --- | --- | --- | --- | --- | --- | --- | --- | --- | --- | --- | --- | --- | --- | --- | --- | --- | --- | --- | --- | --- | --- | --- | --- | --- | --- | --- | --- | --- | --- | --- | --- | --- | --- | --- | --- | --- | --- | --- | --- | --- | --- | --- | --- | --- | --- | --- | --- | --- | --- | --- | --- | --- | --- | --- | --- | --- | --- | --- | --- | --- | --- | --- | --- | --- | --- | --- | --- | --- | --- | --- | --- | --- | --- | --- | --- | --- | --- | --- | --- | --- | --- | --- | --- | --- | --- | --- | --- | --- | --- | --- | --- | --- | --- | --- | --- | --- | --- | --- | --- | --- | --- | --- | --- | --- | --- | --- | --- | --- | --- | --- | --- | --- | --- | --- | --- | --- | --- | --- | --- | --- | --- | --- | --- | --- | --- | --- | --- | --- | --- | --- | --- | --- | --- | --- | --- | --- | --- | --- | --- | --- | --- | --- | --- | --- | --- | --- | --- | --- | --- | --- | --- | --- | --- | --- | --- | --- | --- | --- | --- | --- | --- | --- | --- | --- | --- | --- | --- | --- | --- | --- | --- | --- | --- | --- | --- | --- | --- | --- | --- | --- | --- | --- | --- | --- | --- | --- | --- | --- | --- | --- | --- | --- | --- | --- | --- | --- | --- | --- | --- | --- | --- | --- | --- | --- | --- | --- | --- | --- | --- | --- | --- | --- | --- | --- | --- | --- | --- | --- | --- | --- | --- | --- | --- | --- | --- | --- | --- | --- | --- | --- | --- | --- | --- | --- | --- | --- | --- | --- | --- | --- | --- | --- | --- | --- | --- | --- | --- | --- | --- | --- | --- | --- | --- | --- | --- | --- | --- | --- | --- | --- | --- | --- | --- | --- | --- | --- | --- | --- | --- | --- | --- | --- | --- | --- | --- | --- | --- | --- | --- | --- | --- | --- | --- | --- | --- | --- | --- | --- | --- | --- | --- | --- | --- | --- | --- | --- | --- | --- | --- | --- | --- | --- | --- | --- | --- | --- | --- | --- | --- | --- | --- | --- | --- | --- | --- | --- | --- | --- | --- | --- | --- | --- | --- | --- | --- | --- | --- | --- | --- | --- | --- | --- | --- | --- | --- | --- | --- | --- | --- | --- | --- | --- | --- | --- | --- | --- | --- | --- | --- | --- | --- | --- | --- | --- | --- | --- | --- | --- | --- | --- | --- | --- | --- | --- | --- | --- | --- | --- | --- | --- | --- | --- | --- | --- | --- | --- | --- | --- | --- | --- | --- | --- | --- | --- | --- | --- | --- | --- | --- | --- | --- | --- | --- | --- | --- | --- | --- | --- | --- | --- | --- | --- | --- | --- | --- | --- | --- | --- | --- | --- | --- | --- | --- | --- | --- | --- | --- | --- | --- | --- | --- | --- | --- | --- | --- | --- | --- | --- | --- | --- | --- | --- | --- | --- | --- | --- | --- | --- | --- | --- | --- | --- | --- | --- | --- | --- | --- | --- | --- | --- | --- | --- | --- | --- | --- | --- | --- | --- | --- | --- | --- | --- | --- | --- | --- | --- | --- | --- | --- | --- | --- | --- | --- | --- | --- | --- | --- | --- | --- | --- | --- | --- | --- | --- | --- | --- | --- | --- | --- | --- | --- | --- | --- | --- | --- | --- | --- | --- | --- | --- | --- | --- | --- | --- | --- | --- | --- | --- | --- | --- | --- | --- | --- | --- | --- | --- | --- | --- | --- | --- | --- | --- | --- | --- | --- | --- | --- | --- | --- | --- | --- | --- | --- | --- | --- | --- | --- | --- | --- | --- | --- | --- | --- | --- | --- | --- | --- | --- | --- | --- | --- | --- | --- | --- | --- | --- | --- | --- | --- | --- | --- | --- | --- | --- | --- | --- | --- | --- | --- | --- | --- | --- | --- | --- | --- | --- | --- | --- | --- | --- | --- | --- | --- | --- | --- | --- | --- | --- | --- | --- | --- | --- | --- | --- | --- | --- | --- | --- | --- | --- | --- | --- | --- | --- | --- | --- | --- | --- | --- | --- | --- | --- | --- | --- | --- | --- | --- | --- | --- | --- | --- | --- | --- | --- | --- | --- | --- | --- | --- | --- | --- | --- | --- | --- | --- | --- | --- | --- | --- | --- | --- | --- | --- | --- | --- | --- | --- | --- | --- | --- | --- | --- | --- | --- | --- | --- | --- | --- | --- | --- | --- | --- | --- | --- | --- | --- | --- | --- | --- | --- | --- | --- | --- | --- | --- | --- | --- | --- | --- | --- | --- | --- | --- | --- | --- | --- | --- | --- | --- | --- | --- | --- | --- | --- | --- | --- | --- | --- | --- | --- | --- | --- | --- | --- | --- | --- | --- | --- | --- | --- | --- | --- | --- | --- | --- | --- | --- | --- | --- | --- | --- | --- | --- | --- | --- | --- | --- | --- | --- | --- | --- | --- | --- | --- | --- | --- | --- | --- | --- | --- | --- | --- | --- | --- | --- | --- | --- | --- | --- | --- | --- | --- | --- | --- | --- | --- | --- | --- | --- | --- | --- | --- | --- | --- | --- | --- | --- | --- | --- | --- | --- | --- | --- | --- | --- | --- | --- | --- | --- | --- | --- | --- | --- | --- | --- | --- | --- | --- | --- | --- | --- | --- | --- | --- | --- | --- | --- | --- | --- | --- | --- | --- | --- | --- | --- | --- | --- | --- | --- | --- | --- | --- | --- | --- | --- | --- | --- | --- | --- | --- | --- | --- | --- | --- | --- | --- | --- | --- | --- | --- | --- | --- | --- | --- | --- | --- | --- | --- | --- | --- | --- | --- | --- | --- | --- | --- | --- | --- | --- | --- | --- | --- | --- | --- | --- | --- | --- | --- | --- | --- | --- | --- | --- | --- | --- | --- | --- | --- | --- | --- | --- | --- | --- | --- | --- | --- | --- | --- | --- | --- | --- | --- | --- | --- | --- | --- | --- | --- | --- | --- | --- | --- | --- | --- | --- | --- | --- | --- | --- | --- | --- | --- | --- | --- | --- | --- | --- | --- | --- | --- | --- | --- | --- | --- | --- | --- | --- | --- | --- | --- | --- | --- | --- | --- | --- | --- | --- | --- | --- | --- | --- | --- | --- | --- | --- | --- | --- | --- | --- | --- | --- | --- | --- | --- | --- | --- | --- | --- | --- | --- | --- | --- | --- | --- | --- | --- | --- | --- | --- | --- | --- | --- | --- | --- | --- | --- | --- | --- | --- | --- | --- | --- | --- | --- | --- | --- | --- | --- | --- | --- | --- | --- | --- | --- | --- | --- | --- | --- | --- | --- | --- | --- | --- | --- | --- | --- | --- | --- | --- | --- | --- | --- | --- | --- | --- | --- | --- | --- | --- | --- | --- | --- | --- | --- | --- | --- | --- | --- | --- | --- | --- | --- | --- | --- | --- | --- | --- | --- | --- | --- | --- | --- | --- | --- | --- | --- | --- | --- | --- | --- | --- | --- | --- | --- | --- | --- | --- | --- | --- | --- | --- | --- | --- | --- | --- | --- | --- | --- | --- | --- | --- | --- | --- | --- | --- | --- | --- | --- | --- | --- | --- | --- | --- | --- | --- | --- | --- | --- | --- | --- | --- | --- | --- | --- | --- | --- | --- | --- | --- | --- | --- | --- | --- | --- | --- | --- | --- | --- | --- | --- | --- | --- | --- | --- | --- | --- | --- | --- | --- | --- | --- | --- | --- | --- | --- | --- | --- | --- | --- | --- | --- | --- | --- | --- | --- | --- | --- | --- | --- | --- | --- | --- | --- | --- | --- | --- | --- | --- | --- | --- | --- | --- | --- | --- | --- | --- | --- | --- | --- | --- | --- | --- | --- | --- | --- | --- | --- | --- | --- | --- | --- | --- | --- | --- | --- | --- | --- | --- | --- | --- | --- | --- | --- | --- | --- | --- | --- | --- | --- | --- | --- | --- | --- | --- | --- | --- | --- | --- | --- | --- | --- | --- | --- | --- | --- | --- | --- | --- | --- | --- | --- | --- | --- | --- | --- | --- | --- | --- | --- | --- | --- | --- | --- | --- | --- | --- | --- | --- | --- | --- | --- | --- | --- | --- | --- | --- | --- | --- | --- | --- | --- | --- | --- | --- | --- | --- | --- | --- | --- | --- | --- | --- | --- | --- | --- | --- | --- | --- | --- | --- | --- | --- | --- | --- | --- | --- | --- | --- | --- | --- | --- | --- | --- | --- | --- | --- | --- | --- | --- | --- | --- | --- | --- | --- | --- | --- | --- | --- | --- | --- | --- | --- | --- | --- | --- | --- |

| **Supplementary Table 12.** Neural activation of a whole brain analyses reflecting differences for triple trials comparing the CA+/RA+ group with all other groups. Clusters with a minimum size of k_min_=10^1^ were considered. | | | | | | | |
| --- | --- | --- | --- | --- | --- | --- | --- |
| **Contrast**  **RI_(CS+>CS-)_** | **brain area** | **x** | **y** | **z** | **T** | **p(uc)** | **p(svc_FWE_)** |
| CA+/RA+>[CA-/RA- | superior frontal gyrus (L) | -24 | 42 | 40 | 5.07 | <0.001 | - |
| ,CA-/RA+,CA+/RA-] | precentral gyrus (L) | -32 | 8 | 38 | 4.93 | <0.001 | - |
|  | insula (R) | 36 | -28 | 20 | 4.38 | <0.001 | n.s. |
|  | parietal operculum (R) | 38 | -16 | 20 | 4.07 | <0.001 | - |
|  | inferior temporal gyrus (L) | -58 | -16 | -28 | 4.20 | <0.001 | - |
|  | sup. med. frontal gyrus (R) | 4 | 64 | 20 | 3.87 | <0.001 | - |
|  | parietal operculum(L) | -56 | -6 | 10 | 3.73 | <0.001 | - |
|  | angular gyrus (R) | 46 | -54 | 24 | 3.60 | <0.001 | - |
|  | WM | 22 | -26 | 52 | 4.09 | <0.001 | - |
| CA+/RA+<[CA-/RA-,CA-/RA+,CA+/RA-] | none |  |  |  |  |  |  |

1 = an exception was made for ROIs

n.s. – no suprathreshold voxels

**REFERENCES**

1. Caspi A, Moffitt TE, Thornton A, Freedman D, et al (1996): The life history calendar: A research and clinical assessment method for collecting retrospective event-history data. *Int J Methods Psychiatr Res*. 6: 101–114.
